# Mechanisms Underlying Cancer Chemoresistance: A Starting Point to Identify New Anticancer Strategies

**DOI:** 10.3390/ijms27073122

**Published:** 2026-03-30

**Authors:** Cinzia Domenicotti, Barbara Marengo

**Affiliations:** 1 Department of Experimental Medicine, General Pathology Section, University of Genoa, 16132 Genoa, Italy; 2 IRCCS Azienda Ospedaliera Metropolitana, 16132 Genoa, Italy

Chemoresistance remains a major obstacle in oncology, representing a key factor in treatment failure, disease recurrence, and cancer-related mortality [1,2]. Furthermore, resistance is not a marginal phenomenon, but a central biological property of advanced cancers, since it limits the long-term efficacy of conventional and novel therapeutic approaches. Based on these considerations, this Special Issue aims at providing an overview of some of the biological processes that allow tumor cells to survive therapeutic pressure. The contributions highlight how chemoresistance is the result of a dynamic interplay between genetic alterations, metabolic rewiring, stress adaptation pathways, and tumor microenvironmental constraints, rather than a monofactorial mechanism.

A more frequently discussed theme in this collection of articles is the key role of ATP-binding cassette (ABC) transporters, which are cellular transport systems that not only facilitate drug efflux but also play a crucial role in lipid transport and homeostasis [3,4]. In this regard, the minireview by Alketbi’s group (contribution 1) is focused on the role of ABCA transporters in colorectal cancer (CRC) progression and therapy resistance, suggesting the possibility to utilize them as potential diagnostic and/or therapeutic targets. In fact, ABCA members, overexpressed in cancer cells, are involved in cholesterol transport which, via Wnt-, Hedgehog-, and TGF-β-dependent signaling pathways, can control the response of epithelial–mesenchymal transition (EMT), promoting tumor aggressiveness and resistance (contribution 1).

In CRC, tumor progression has been linked to dysregulation of the lipid profile, since lipids play a crucial role not only in membrane formation but also in supporting efficient energy metabolism and promoting tumorigenesis through the activation of several signaling cascades [5].

The role played by ABC transporters was also investigated in the article by Stefanski and co-authors, who demonstrated that loss of Adenomatous Polyposis Coli (APC) induces doxorubicin (DOX) resistance by increasing the activity of multidrug resistance protein 1 (MDR1) and multidrug resistance-associated protein 1 (MRP1) in human breast cancer cell lines and patient samples (contribution 2). Herein, the authors also demonstrated in mouse mammary tumor cells that the pharmacological inhibition of MDR1 or silencing of MRP1 is able to decrease the population of tumor-initiating cells (TICs) and increase DOX-induced cell death. In this regard, the authors emphasize the importance of understanding the direct role of APC loss in regulating cancer stem cells in order to bypass chemoresistance [6]. Therefore, the authors suggest that targeting either upstream regulators of ABC transporters (i.e., STAT3, miR-200C, mir-124-3P) or TIC populations could offer a new therapeutic perspective in chemoresistant APC-deficient cancers.

In this context, the article by Stasiak et al. deals with the action of elacridar (GG918 and GF120918), a potent third-generation inhibitor of glycoprotein-P (P-gp; MDR-1) which has the ability to reverse MDR in paclitaxel-resistant ovarian cancer (PAC) cell lines (contribution 3). Although elacridar has proven effective in inhibiting P-gp activity and restoring sensitivity to some drugs in two-dimensional cultures, its limited efficacy in three-dimensional spheroid models highlights the critical influence of tumor architecture and microenvironmental factors on drug response. Three-dimensional systems better recapitulate the pathophysiological conditions of tumor tissue and its interactions with the microenvironment, which cannot be reproduced in 2D models. However, reproducing a 3D chemoresistant cancer model is extremely challenging, since factors such as drug diffusion, cell density, interactions between cancer cells and other cells of the tumor microenvironment, the expression of extracellular matrix molecules, and MDR-related genes are involved. This limitation is highlighted in translational oncology studies and underscores the pressing and justified need to routinely use advanced preclinical models, such as 3D models, to better understand the mechanisms underlying chemoresistance and, consequently, to easily predict the clinical outcomes of treatments [7,8].

As recently reviewed [9,10], chemoresistance is due to several key mechanisms, including abnormal drug efflux, metabolic reprogramming, evasion of apoptosis, autophagy, activation of cancer stem cells, and increased DNA repair. In this collection of articles, Flasarova’s group analyzed the gene expression of proteins involved in DNA repair in ovarian cancer patients treated with cytoreductive surgery and hyperthermic intraperitoneal chemotherapy (HIPEC) (contribution 4). This study provides clinically relevant information on how modulation of DNA repair pathways correlates with tumor survival and metastatic progression. In particular, significant gene interactions were found among *CCNH*, *XPA*, *SLK*, *RAD51C*, *XPA*, *NEIL1*, and *ATR* in primary tumor tissue and among *ATM*, *ATR*, *BRCA2*, *CDK7*, *MSH2*, *MUTYH*, *POLB*, and *XRCC4* in metastases (contribution 4). Furthermore, an important correlation was identified between gene expression and overall survival (OS), demonstrating that low expression of these DNA repair genes correlates with worse OS. Based on these findings, it can be argued that the integration of DNA repair gene profiles into the clinical management could enable patient stratification, allowing for personalized cancer treatment.

Another contributing factor to chemoresistance is represented by the metabolic plasticity that characterizes different types of cancer. Metabolic reprogramming is considered a hallmark of cancer and includes changes in glucose, amino acids, and lipid metabolism that are utilized by cancer cells to continue growing and surviving [11,12]. In this context, the review by Bingham and Zachar (contribution 5) provides evidence of a continuous rewiring of lipid metabolism in tumor cells as they progress toward more advanced and therapy-resistant cancers. This redesigned lipid metabolism, termed the lipid metabolism resistance system (LMRS), allows tumor cells to control the catabolic flux of fatty acids based on oxygen availability [13]. This lipid reprogramming includes a high capacity for de novo fatty acid synthesis, an increased ability to actively import fatty acids from the surrounding environment, and greater access to the lipids thanks to a reciprocal interaction with stromal cells. The LRMS process is characterized by three major steps consisting of (i) peroxisomal fatty acid beta-oxidation initiated by acyl-coenzyme A oxidases (Acox), (ii) desaturation of saturated fatty acids, commonly catalyzed by stearoyl-CoA desaturase (SCD1), leading to a monounsaturated fatty acid (MUFA), and (iii) mitochondrial complete oxidation of the products of the two steps to CO_2_ and H_2_O_2_.

A large body of evidence supports the role of LMRS in advanced carcinoma therapy resistance, suggesting that targeting the LMRS pathway may sensitize cancer cells to currently used therapeutic agents. It is worth noting that preclinical studies report the repurposing of some FDA-approved drugs as compounds capable of targeting lipid catabolic pathways and acting as adjuvants to standard treatments in in vitro and in vivo cancer models [14,15].

Among the multifaceted spectrum of mechanisms that enable tumor cells to withstand the most potent therapeutic strategies, the activation of autophagy represents a critical factor and a key hallmark of cancer [11]. Autophagy is a self-catabolic process characterized by the degradation of proteins, cellular macromolecules, and organelles in a lysosomal-dependent pathway [16]. In cancer, autophagy exhibits a dual role since it can promote cell survival and facilitate cell death, depending on the context [17,18]. In the present collection of articles, Lai and co-authors found that Unc-51-Like Autophagy Activating Kinase 2 (ULK2), a key pro-autophagy protein, is overexpressed in chemoresistant FLT3-mutated acute myeloid leukemia (AML) cells (contribution 6). In fact, ULK1/2 inhibition was able to sensitize FLT3-mutated AML cells to cytarabine and prevent relapse in vitro. In agreement with this evidence, clinical and preclinical studies have demonstrated that autophagy is a key mediator of therapy tolerance and minimal residual disease [19,20]. Furthermore, the higher expression of ULK2 in relapsed cancer patients further strengthens the clinical relevance of autophagy and encourages the development of selective inhibitors to use in combination therapy regimens.

Overall, the articles that we have selected in this Special Issue support the fact that cancer chemoresistance is a multifactorial process that could be counteracted only by a multidisciplinary approach [21]. Notably, cancer drug resistance can be considered a Darwinian evolution to a resilient tumor cell population capable of withstanding different therapeutic pressures [22]. Moreover, this adaptive process has been compared to antimicrobial drug resistance, resulting from the ability of pathogens to evade the immune system and antimicrobial agents [21].

Taking into consideration the multiple mechanisms of cancer chemoresistance [23], combination therapies targeting different resistance hallmarks may enhance cancer cell killing and reduce the probability of developing drug resistance. Looking into the future, multidisciplinary studies are needed to (i) integrate functional assays with molecular profiling in order to identify clinically relevant resistance hallmarks; (ii) identify resistance biomarkers to improve patient stratification; and (iii) develop and utilize physiologically relevant experimental models to bridge the gap between preclinical discovery and clinical application.

As we conclude this editorial project, we wish to thank all the authors who contributed to this Special Issue and shared their recent discoveries. Finally, we would like to thank the reviewers for their guidance and the editorial team for their kind support.
